# Development of a New Branded UK Food Composition Database for an Online Dietary Assessment Tool

**DOI:** 10.3390/nu8080480

**Published:** 2016-08-05

**Authors:** Michelle C. Carter, Neil Hancock, Salwa A. Albar, Helen Brown, Darren C. Greenwood, Laura J. Hardie, Gary S. Frost, Petra A. Wark, Janet E. Cade

**Affiliations:** 1 Nutritional Epidemiology Group, School of Food Science and Nutrition, University of Leeds, Leeds LS2 9JT, UK; n.hancock@leeds.ac.uk (N.H.); ml09saa@leeds.ac.uk (S.A.A.); h.c.e.brown@leeds.ac.uk (H.B.); j.e.cade@leeds.ac.uk (J.E.C.); 2 Department of Food Science and Nutrition, King Abdul-Aziz University, PO Box 42807, Jeddah 21551, Saudi Arabia; 3 Division of Biostatistics, Leeds Institute for Cardiovascular and Metabolic Medicine, University of Leeds, Leeds LS2 9JT, UK; d.c.greenwood@leeds.ac.uk; 4 Molecular Epidemiology Group, Leeds Institute for Cardiovascular and Metabolic Medicine, University of Leeds, Leeds LS2 9JT, UK; l.j.hardie@leeds.ac.uk; 5 Nutrition and Dietetic Research Group, Department of Investigative Medicine, Hammersmith Hospital, Imperial College London, London W12 0NN, UK; g.frost@imperial.ac.uk; 6 Global eHealth Unit, Department of Primary Care and Public Health, School of Public Health, Imperial College London, London W6 8RP, UK; p.wark@imperial.ac.uk

**Keywords:** food composition database, dietary assessment, nutrition assessment

## Abstract

The current UK food composition tables are limited, containing ~3300 mostly generic food and drink items. To reflect the wide range of food products available to British consumers and to potentially improve accuracy of dietary assessment, a large UK specific electronic food composition database (FCDB) has been developed. A mapping exercise has been conducted that matched micronutrient data from generic food codes to “Back of Pack” data from branded food products using a semi-automated process. After cleaning and processing, version 1.0 of the new FCDB contains 40,274 generic and branded items with associated 120 macronutrient and micronutrient data and 5669 items with portion images. Over 50% of food and drink items were individually mapped to within 10% agreement with the generic food item for energy. Several quality checking procedures were applied after mapping including; identifying foods above and below the expected range for a particular nutrient within that food group and cross-checking the mapping of items such as concentrated and raw/dried products. The new electronic FCDB has substantially increased the size of the current, publically available, UK food tables. The FCDB has been incorporated into myfood24, a new fully automated online dietary assessment tool and, a smartphone application for weight loss.

## 1. Introduction

The ability to accurately estimate dietary intake is fundamental to nutritional epidemiology. Traditionally, dietary consumption data is collected by paper based methods such as food frequency questionnaires, food diaries and interviewer administered 24-h recalls. Using a paper based method, the researcher is required to “code” dietary intakes in order to provide data on energy and nutrient intake. The coding process is aided by a food composition database (FCDB) which usually contains country-specific, detailed information on the nutritional composition of foods [1]. 

The current edition of the UK food composition tables contains ~3300 mostly generic food and drink items [2]. The tables are also available in electronic format as an integrated dataset [3]. An electronic FCDB has potential to automate the often time consuming and costly food consumption coding process given that respondents can enter their dietary intake directly. There are a number of existing automated and online dietary assessment tools which incorporate electronic FCDBs [4,5,6,7]. 

In the UK, a fully automated online 24-h dietary assessment system, myfood24 (measure your food on one day) has been developed [8]. An automated system such as myfood24 places responsibility on the respondent (rather than a trained nutritionist) to correctly identify and select the appropriate food or drink item that they have consumed. This presents a unique challenge, as the electronic FCDB must sufficiently reflect the expanding range of foods available to the British consumer, and present food descriptions in a user friendly and accessible way. 

To better reflect the range and diversity of food products available in the UK and to meet the needs of the myfood24 dietary assessment tool, a new comprehensive electronic FCDB has been developed (hereafter referred to as the “myfood24 FCDB”). Here, we aim to report on the development of the myfood24 FCDB and considerations around quality control and database maintenance. 

## 2. Materials and Methods 

**Figure 1:**
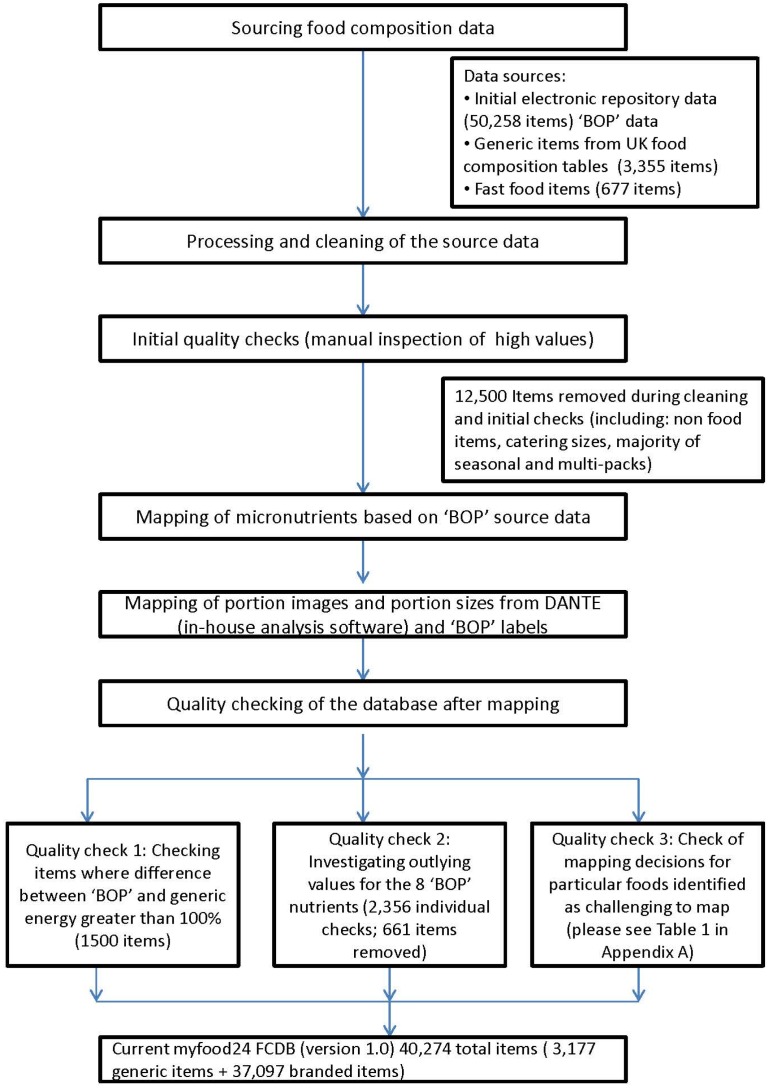
Flow chart of the myfood24 electronic food composition database development process. “BOP” = Back of Pack.

Figure 1 presents a flow chart of the database development process.

### 2.1. Sourcing of Food Composition Data

The myfood24 FCDB (version 1.0, 2016, University of Leeds, Leeds, UK) was created in Microsoft Access and constructed from a number of data sources, including: (1) generic items from the 2002 McCance and Widdowson’s The Composition of Foods integrated dataset (3355 items) [9]; (2) “Back of Pack” (BOP) data provided freely by a company which holds a large, electronic repository (50,258 in the initial extract); and (3) fast food items (677 items), the majority of which were sourced from manufacturer data published online and in rare instances directly from the manufacturer. Data for BOP macronutrients; energy (kcal), protein (g), fat (g), saturated fat (g), carbohydrate (g), total sugars (g), Association of Official Analytical Chemists (AOAC) fibre (g) and sodium (mg) included both branded and supermarket own brand food and drink items. 

In the UK, the BOP nutrient information is legally required to be the “average value” defined as the “value that best represents the amount of the nutrient which a given food contains, and allows for natural variability of foodstuffs, seasonal variability, patterns of consumptions and other factors which may cause the actual value to vary [10]”.

The “average values” on BOP are derived from one of three methodologies: (1) the manufacturers direct chemical analysis of the food; (2) a calculation from the known or actual average value of the ingredients used; or (3) a calculation from generally established and accepted data [2]. The EU FIC guidance [10] includes tolerance levels which manufacturers must abide by and rounding guidelines for the amount of nutrients that can be regarded as negligible and declared as “0 g” or as “<*X* g” on the nutrition labeling [11]. Table 1 shows the tolerances for the BOP nutrient data included in the myfood24 FCDB as defined by the EU FIC regulation [10].

### 2.2. Cleaning and Processing of the “Back of Pack” Macronutrient Data 

The initial 50,258 BOP extract provided from the commercial electronic repository was thoroughly quality checked and cleaned. This involved: (1) Removing 1982 non-food items, such as cigarettes, medicines and baby products; (2) Populating missing data for individual BOP nutrients (where a branded item did not have values for all 8 BOP nutrients, values were taken from the UK food tables for similar items. In these cases, the mapping was done by matching based on item description alone. The majority of these items were alcoholic beverages for example, which are not legally required to carry a food label in the UK); (3) Reformatting the data for use in the myfood24 online dietary assessment tool; (4) Manually inspecting for and correcting spurious values (for example, inspecting serving size field for decimal points placed incorrectly); (5) Removing multi-pack and catering size items; (6) Removing the majority of seasonal celebration items due to their limited shelf life and likely lack of availability in subsequent years. 

The generic items from the UK food composition tables [9] were also cleaned, this involved: (1) Removal of items thought to be unlikely to be consumed by the adult UK population (such as human breast-milk and infant formula milk); and (2) Simplification and rewording of descriptions to make them more logical and accessible to a non-nutritionist (i.e., “milk, semi-skimmed, pasteurised, average” changed to “milk, semi-skimmed”).

Initial quality checks were conducted on the whole database rather than checking each data source separately. Food items with very high values were identified and manually inspected to confirm that the top food contributors to the nutrient were as expected. The following cut-off points were applied: energy (≤900 kcal/100 g) top contributors checked to be fats and oils; total carbohydrate (≤99.9 g/100 g), top contributors checked to be pure table sugar and sweets; protein (≤89 g/100 g), top contributors checked to be protein supplements; sugar (≤100 g/100 g), top contributors checked to be table sugar; total fat (≤99.9 g/100 g), top contributors checked to be fats and oils; saturated fat (≤86 g/100 g), top contributors checked to be coconut oil products and butter; fibre (≤54 g/100 g), top contributors checked to be dried herbs; salt (≤39 g/100 g), top contributors checked to be salt mixes, sauces and seasoning. Most corrections were made to sodium where the most common error appeared to be a data entry error at the source of the original data (i.e., a decimal point was placed incorrectly or salt and sodium content were swapped). The highest 400 sodium records were manually checked for accuracy. More comprehensive quality checking procedures were applied following micronutrient mapping. During the initial processing and cleaning, 12,500 items were removed from the extract provided by the electronic repository.

Additional database formatting was conducted in order to enhance the search function of the myfood24 dietary assessment tool [8]. Formatting tasks included: (1) Amending food descriptions to ensure search results displayed more popular foods first. For example, a search for milk in the myfood24 FCDB would return “milk pudding” near the top of the list so the descriptor was changed to “pudding, milk”. (544 descriptions changed); (2) Common synonyms (e.g., “coke” appended to “coca-cola”) and potential misspellings added to the database to aid searching (30,733 synonyms and misspellings added in total); (3) Serving size description added where necessary to clarify serving unit (e.g., for powdered foods servings might be “as made up” or “as powder”); and (4) Common accompaniment foods (i.e., milk with tea) added (the myfood24 tool offers prompts for certain common accompaniments to foods). 

The cleaned macronutrient BOP data was used as a foundation for linking additional information, including: micronutrient values, generic food and drink items and portion sizes and images (to be discussed in more detail in the Section 2.5.

### 2.3. “Mapping” of Micronutrients to Macronutrient BOP Data 

In order to build an electronic FCDB comprising both macronutrient and micronutrient data, a “mapping exercise” was conducted. The purpose of the mapping exercise was to match food and drink items based on the description and BOP macronutrient data to appropriate generic food codes in order to populate micronutrient data. Generic food codes were used from the 2002 McCance and Widdowson’s The Composition of Foods integrated dataset [9], (the more recent 7th edition of the UK food tables was not available at that time) [2]. 

**Figure 2:**
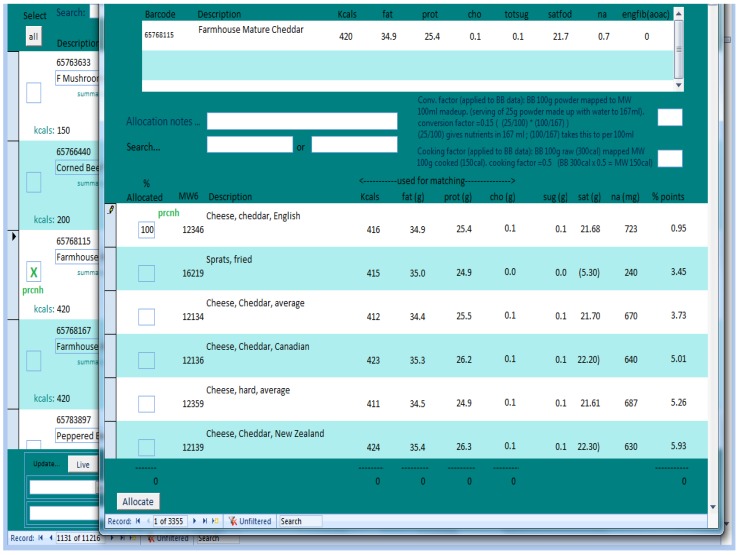
The semi-automated system for mapping branded foods from the BOP nutrition data to the most appropriate generic food. Sprats are showing in this list because, based on the mapping algorithm, they were nutritionally similar to the branded products on the 4 macronutrients. The nutritionist coding made the final decision which food in the list was most appropriate to map to and in what proportion.

Given the number of items to match, a program was developed to semi-automate the mapping exercise. Figure 2 shows the semi-automated program for mapping a single branded food item based on its BOP nutrient data to a generic code in order to populate micronutrient information.

The mapping exercise involved matching foods based on ranking by percentage agreement on energy, fat, protein and carbohydrate. For single branded items which would be mapped to one single generic item, the program presented the sum of the percentage difference on four of the BOP nutrients (energy, fat, protein and carbohydrate) and ranked them in ascending order so that the best matches appeared first. The matching program suggested a range of generic food options for mapping to a particular branded product and the nutritionist was able to make a decision to select the most appropriate option or reject it and search manually. A generic food is selected by specifying a percentage allocated value, enabling several generic foods to be mapped to a branded item. For branded items where it was likely two or more generic food codes would need to be combined (e.g., breaded fish), the system compared branded items with 2 lists of generic items limited by food group. The program then worked through all combinations of generic items from each food group from 0% through 100% match in 10% increments. The UK food tables [9] are limited in terms of ready meals and convenience foods, so many branded items had to be broken down into their constituent ingredients and coded as a recipe. In these cases it was often guided by the “ingredients list” (if available) detailed on the BOP food label. 

In circumstances where a particular food or drink item was not available in the UK food composition tables, the nearest sensible alternative in terms of nutrient content was chosen based on the nutritionists expert knowledge (for example, premixed spirit based drinks with relatively low alcohol content such as “Bacardi Breezers” which are not present in the 6th edition of the UK food tables were proportionally mapped to a combination of 90% of the “alcoholic spirits” code and 10% of the “carbonated fruit juice” code). Branded fresh fruit and vegetables were removed in order to streamline the appearance of the search list in the myfood24 tool. As a result, if a user wants to select a pre-packaged bunch of bananas, for example, they would need to input individual generic bananas. 

Table A1 highlights some of the specific mapping decisions that were made. It is worth highlighting that the mapping exercise populated micronutrient values; the macronutrients for all branded foods in the myfood24 FCDB are the values taken from the BOP food label (where populated). A minority of items provided by the electronic repository did not contain BOP data; in these cases, both macronutrient and micronutrient data was populated by an appropriate generic code based on food item name and/or description. The majority of these items were alcoholic beverages which are not legally required to carry a food label in the UK. 

### 2.4. Portion Size Estimation 

Food portion images were obtained from the Young Person’s Food Atlas Secondary publication, created for the Food Standards Agency by researchers at Newcastle University [13]. The foods included in the Atlas are the top 100 foods in terms of frequency of consumption, weight of consumption and contribution to energy intake, identified from data collected during the National Diet and Nutrition Survey conducted with young people (4–18 years) [13]. The food portion images chosen to be used in myfood24 comprise 409 images covering 59 food types. Some images were used for similar food items, for example, the image for sliced chicken breast was also applied to other white sliced meats, such as turkey and pork. Therefore in total, 5669/40,274 (14%) food and drink items have associated portion images. Each food type with associated portion images has the option for the user to select from seven portion size images. 

For both generic and branded food, portion sizes were obtained from the Food Standards Agency’s Food Portion Sizes publication [14]. In addition, to fill in gaps where portion sizes were absent from the Food Standards Agency publication, average serving sizes were taken from dietary data coded in DANTE (Diet and Nutrition Tool for Evaluation). DANTE is an in-house Microsoft Access based food diary analysis program developed by the Nutritional Epidemiology Group at the University of Leeds, Leeds, UK. When coding food diaries in DANTE the coder is able to manually input a portion size if it has been specified in the food diary. A DANTE serving size was created by taking an average of portion sizes entered into DANTE during previous UK dietary surveys. All of the DANTE serving sizes created were manually checked by a nutritionist against similar foods in the Food Standards Agency publication to confirm that they were sensible. For branded items, pack sizes (where available from the electronic FCDB, and appropriate) were also included as a portion estimate option. Multi-pack items were deleted where the single item was also available. For a multi-pack food item, the multi-pack weight was divided by the number of items and used as a portion option with a description, “per item”.

### 2.5. Quality Checking of the Myfood24 FCDB 

Three rounds of quality checks were performed on the myfood24 FCDB after completion of the micronutrient mapping exercise: (1) preliminary checks to identify mapped foods where the difference between the BOP and generic energy value (kcal/100 g) was greater than 100%. The decision to use 100% was pragmatic in order to give an initial assessment of the scale of error before moving to more detailed checks; (2) a detailed check of outlying values for the 8 BOP nutrients; (3) a check of mapping decisions made for particular food types which were identified as the most challenging to map (i.e., where there are volume changes on cooking or where there are few appropriate items for mapping in the UK food tables). This involved a nutritionist checking that mapping was consistent and in accordance to the protocol for the food types listed in Table A1 and remapping items if necessary. The two detailed quality checks (steps 2 and 3 above) were performed by two nutritionists respectively who were not involved in the original mapping exercise. 

In order to identify outlying values for each of the 8 BOP nutrients, a top and bottom cut-off value was used within each myfood24 food group and for each nutrient. The cut-off points were the minimum and maximum range within each myfood24 food group and for each BOP nutrient from the most recent version of the UK food composition tables [2]. There are 19 myfood24 food groups and 15 food groups in the UK food composition tables and whilst some are directly equivalent, the food groups in the myfood24 FCDB are not identical. Pragmatic decisions were made to match food group categories during this process to identify the appropriate cut-offs. For example, in myfood24 there is a “frozen foods” group which does not have an equivalent in the UK food tables so all foods from the UK food tables containing the word “frozen” were identified and used to determine the lower and upper range for each BOP nutrient within this food group. 

Food products with nutrients identified as outliers were checked against available manufacturer data (online, by contacting manufacturers directly or visiting supermarkets). An exception was the fast food data which was not cross-checked in this way as it had already been sourced from the manufacturer’s website. There were three outcomes for the product upon checking: (1) If no manufacturer information could be found for the product it was assumed that it had been discontinued and the item was removed from the database; (2) If the nutrient value in the manufacturer information differed from the value identified as an outlier it was assumed that the product had been reformulated and the value was corrected in the myfood24 FCDB; The exception were instances where the manufacturer data appeared implausible and was likely to be an error in the food label and (3) No changes were made if we identified an outlier to be plausible, i.e., if the manufacturer corroborated the database value or the manufacturer’s data were likely an error in the food label as they appeared implausible. The outcome of these quality checks is detailed in the results section.

## 3. Results

In total, version 1.0 of the myfood24 FCDB contains 40,274 branded and generic food items with both macronutrient and micronutrient data. The items contained in the myfood24 FCDB were chosen to reflect the foods available to the UK population. The supermarket Tesco was the first supermarket to be incorporated into the myfood24 FCDB as it is the largest in the UK with 3535 stores nationwide [15] and has the largest market share (28.4%) [16]. The myfood24 FCDB also contains food products from the most popular pre-packed food brands. Table 2 shows the number of food products in the myfood24 FCDB by brand penetration for the 20 most popular brands consumed in the UK (according to market penetration as of 2014 [17]).

Table 3 shows the number of food and drink items mapped within each food group and the percentage agreement in each category between the generic and back of pack nutrient data for energy (kcal). Over 50% of items were individually mapped to within 10% agreement with the generic food item for energy. The majority of foods (80%) were mapped to a single generic item; with multiple generic items for the rest. For more complex recipes (such as ready meals) and where ingredient lists were available on the food label, the nutritionist would allocate individual foods manually to create the recipe. For example, one ready prepared korma curry was mapped to 23 ingredients using the ingredient list on the food label. 

The largest food groups mapped were “cakes, biscuits, chocolates and other snacks” (6918 items, 18% of the database); “alcoholic drinks” (5692 items, 15%) and “sauces and condiments” (3635 items, 9%). The “soft drinks” food group in the myfood24 FCDB has the largest mean % difference in energy between the BOP and generic items (18%).

### Results of the Quality Checking of the Myfood24 FCDB 

After an initial check, 1500 foods were identified as having an energy difference between branded and generic items of greater than 100% with 168 having a greater than 1000% energy (kcal) difference. All of these foods were manually checked. The majority of these items were “diet” products (i.e., diet soft drinks, reduced fat mayonnaise, reduced sugar frozen desserts) or protein supplements. Diet products have been mapped to generic “non diet” codes as there were no suitable alternatives (for example, “Coca-Cola Zero” has been mapped to a generic “cola” code). As the myfood24 FCDB uses data for the 8 BOP nutrients (i.e., for “Coca-Cola Zero” the sugar content is taken from the food label rather than the generic “cola” code) with micronutrient data appended, the diet products were responsible for this apparently large difference in energy intake between branded and generic products. As the BOP nutrients were accurate these products did not need to be remapped. The remainder of the mapping error at this stage was for concentrated products. All of the concentrated products such as powdered desserts and dilute drinks were checked again and remapped if a mapping error was identified. 

Food products with nutrients identified as outliers were checked against available manufacturer data (online, by contacting manufacturers directly or visiting supermarkets). There were three outcomes for the product upon checking: (1) If no manufacturer information could be found for the product it was assumed that it had been discontinued and the item was removed from the database; (2) If the nutrient value in the manufacturer information differed from the value identified as an outlier it was assumed that the product had been reformulated and the value was corrected in the myfood24 FCDB. The exception were instances where the manufacturer data appeared implausible and was likely to be an error in the food label; and (3) No changes were made if we identified an outlier to be plausible, i.e., if the manufacturer corroborated the database value or the manufacturer’s data were likely an error in the food label as they appeared implausible.

In total, across the food groups and nutrients, 22,356 individual nutrient checks were made on foods identified as outliers (often the same food appeared as an outlier for several of the 8 BOP nutrients). Of these 22,356 outlying values, 17,217 appeared to be corroborated by manufacturer data (and plausible for the particular nutrient in that food type) so were left unchanged in the database. Updates were made in the database to 3075 nutrient values to reflect currently available manufacturer data. No manufacturer data was available for 2516 nutrient values and these foods were hidden in the live database. As often a single item would have all of a number of nutrient values missing this actually equated to a total of 661 food and drink items being removed from the database. 

## 4. Discussion

A new comprehensive UK FCDB has been developed for incorporation into an online dietary assessment tool, myfood24 [8]. The database has also been incorporated into “My Meal Mate”, a smartphone application for weight loss [18]. Version 1.0 of the new database contains 40,274 generic and branded items with associated 120 macronutrient and micronutrient data and 5669 items with portion images. This database has increased the size of the current UK food composition tables by tenfold with the inclusion of branded food products. A micronutrient mapping exercise has been conducted to match food and drink items based on their description and BOP nutrient data to generic food codes. This mapping process has provided a comprehensive macronutrient and micronutrient UK FCDB. The myfood24 FCDB development process, including sourcing the food composition data and cleaning and mapping of micronutrients to back of pack (BOP) data, took approximately 18 months, involving a small team of nutritionists, a dietitian, data entry assistants and a database manager. Management, quality checking and updating of the database is an ongoing process. 

A challenge in the ongoing management of the myfood24 FCDB is keeping it up to date. The food and drink industry is the largest manufacturing sector in the UK [19] and invests substantially in research and development. An estimated 10,000 new food and drink products are introduced each year and other products are discontinued as retailers react to changes in demand [19]. Many of these products might only be available for a very short time and sometimes just for a matter of days if they are for seasonal occasions. Nutrient values in established products also change due to product reformulation. 

We plan to update the database at regular intervals. There are also plans to update the myfood24 FCDB by re-mapping the branded products to the most recent version of the UK food composition tables [2]. 

One approach to maintaining an up to date FCDB is “crowdsourcing”, whereby members of the public are given the ability to add in foods and nutrient values. “For example, the ‘FoodSwitch’ app developed by researchers in Australia, uses crowdsourcing of photographs taken by users (front of package, ingredients list and nutrient information panel) which are then quality checked by a data management centre [20]”. This approach allows a FCDB to grow very large. For example, some commercial FCDB’s such as that contained within “diet tracking” apps like “myfitnesspal” report database sizes of over 100,000 items [21]. However, the quality of a crowdsourced database is not always known. A recent study investigating food diaries collected by 23 different smartphone applications for weight loss found the accuracy of energy intake to be variable when compared to a 3 day weighed food record [22]. Whilst the mean difference between the apps and the weighed food record was relatively small (i.e., 127 KJ; 95% CI −45 to 299) the difference in energy reported ranged from −700 KJ to 1001 KJ. Many of the apps in that study maintained a large database through crowdsourcing, and the results shows the variability in energy reporting using electronic food databases from different sources [22]. In order to ensure database quality, updates to the myfood24 FCDB will be done in-house rather than by crowdsourcing at this moment in time. 

With regard to the agreement in terms of energy (kcal) between the generic and mapped values, the largest difference was seen for the “soft drinks” myfood24 food group. Most of this difference was found to be due to “diet soft drinks”, as they had been mapped to a generic “soft drink” code as no sugar free alternative was available. As the myfood24 FCDB uses data for the 8 BOP nutrients with micronutrient data appended, the “diet” soft drinks were not remapped. In the past, coders have had to allocate foods such as this where no suitable generic code was available to an alternative, such as in the case of diet drinks “water”. Any results derived from such coding decisions would be affected by these selections.

### 4.1. Strengths and Limitations of the Myfood24 FCDB

Providing both branded and generic food options, the myfood24 FCDB represents a new resource for use in the myfood24 online dietary assessment tool. The BOP macronutrient data for branded products has potential to more accurately represent dietary intake and improve nutritional assessment. Further research is necessary to determine the impact of a respondent choosing branded foods or generic food and whether this makes a substantial difference to estimates of dietary intake. 

Although a very large database, not all major UK supermarkets are represented within version 1.0 of the myfood24 FCDB. There is still a need for further expansion in order to offer more choice to the user. Version 1.0 of the myfood24 FCDB contains >4500 items from Tesco but other major UK supermarkets such as ASDA and Co-Op are being prepared to be added. Although thorough quality checking has been conducted, there is still potential to introduce error at a number of stages during creation of the FCDB, for instance: (1) The BOP data supplied to the electronic repository may have errors; (2) Data entry error may be introduced when the BOP data is added to the repository; (3) Error may be introduced by incorrectly mapping branded food items to generic entries (although checking mapping for groups identified as particularly challenging to map such as concentrated products) may have mitigated some of this; (4) The product may be reformulated so that the FCDB values are incorrect. 

### 4.2. Future Plans 

Further expansion of the myfood24 FCDB is planned to include a wider range of UK supermarket own brand data. There is also a plan to repeat the “mapping exercise” to re-map foods to the latest version of the UK food composition tables [2]. The structure of the myfood24 dietary assessment tool allows for different electronic FCDB’s to be easily “plugged in” and for the current UK database to be updated. International versions of the myfood24 tool are in preparation including an Australian and German version. In order to keep the myfood24 FCDB up to date and to continue to host the myfood24 tool, there are plans to commercialise it. 

## 5. Conclusions 

This paper has described the development of a new comprehensive UK FCDB which has been developed for incorporation into an online dietary assessment tool, myfood24. Version 1.0 of the myfood24 FCDB database contains 40,274 generic and branded items with associated 120 macronutrient and micronutrient data and 5669 items with portion images. This database has increased the size of the current UK food composition tables by tenfold with the inclusion of branded food products. Micronutrient data has been appended to “Back of Pack” nutrient data for branded products by a “mapping exercise”. There is potential for improving dietary assessment with a detailed branded food database. The myfood24 FCDB represents a new resource but there remains a challenge to keep it up to date and to fully reflect the large number of branded products available to the UK consumer. 

## Figures and Tables

**Table 1:** Tolerances for the nutrition declaration on foods (other than food supplements) and declarations for negligible amounts specifically for BOP nutrients.

Nutrient	Tolerances for Foods (Includes Uncertainty of Measurement)	Negligible Amount	Nutrition Declaration
Vitamins	+50% *–35%	N/A	N/A
Minerals	+45%–35%	N/A	N/A
Carbohydrates, Sugars, Protein	<10 g per 100 g: ±2 g	No detectable amount is present or concentration is ≤0.5 g per 100 g or per 100 mL	0 g or <0.5 g
	10–40 g per 100 g: ±20%		
	>40 g per 100 g: ±8 g		
Fibre	<10 g per 100 g: ±2 g	N/A	N/A
	10–40 g per 100 g: ±20%		
	>40 g per 100 g: ±8 g		
Fat	<10 g per 100 g: ±1.5 g	No detectable amount is present or concentration is ≤0.5 g per 100 g or per 100 mL	0 g or <0.5 g
	10–40 g per 100 g: ±20%		
	>40 g per 100 g: ±8 g		
Saturates	<4 g per 100 g: ±0.8 g	No detectable amount is present or concentration is ≤0.1 g per 100 g or per 100 mL	0 g or <0.1 g
	≥4 g per 100 g: ±20%		
Mono-unsaturates, Polyunsaturates	<4 g per 100 g: ±0.8 g		
	≥4 g per 100 g: ±20%		
Sodium	<0.5 g per 100 g: ±0.15 g	N/A	N/A
	≥0.5 g per 100 g: ±20%		
Salt	<1.25 g per 100 g: ±0.375 g	No detectable amount is present or concentration is ≤0.0125 g per 100 g or per 100 mL	0 g or <0.01 g
	≥1.25 g per 100 g: ±20%		

* For vitamin C in liquids, higher upper tolerance values could be accepted. Sources [12] for data on tolerances for foods [10] and for rounding based on negligible amount [11].

**Table 2:** Top 20 UK food and drink brands in terms of market penetration and number of barcoded items available within the brand in the myfood24 electronic food composition database.

Brand	Market Penetration (%) *	Number of Barcoded Items in the Myfood24 FCDB
Heinz	91	280
McVitie’s	88	67
Warburtons	86	76
Birds Eye	78	148
Kingsmill	76	45
Hovis	74	55
Walkers	74	105
Cadbury Dairy Milk	73	126
Princes	72	214
Jacob’s	67	138
Young’s	65	116
McCain	63	74
Aunt Bessie’s	63	72
Müller	63	70
Fox’s	62	80
Bisto	62	44
Coca-Cola	61	7
Kit Kat	59	18
Maltesers	58	11
Mr Kipling	58	69

* Penetration (%) Number of UK households that buy brand/households surveyed by Kantar World Panel. Data from 412,000 household panels, the penetration represents how real shoppers purchased foods in 2014. Source: Brand information from Kantar World Panel “Brand footprint report” [17].

**Table 3:** The number of food and drink items mapped within each food group and mean % difference in each category between the generic and back of pack nutrient data for energy (kcal).

Category	Count	% of Total Database	Mean % Difference in kcal between Generic vs. BOP)
Cakes, biscuits, chocolates & other snacks	6918	18	3
Alcoholic drinks	5692	15	−2
Sauces and condiments	3635	9	9
Dairy and eggs	3596	9	0
Ready meals, quiches, pizza, pasta, soup	3315	9	1
Bread and grains	2387	6	5
Meat and poultry	1952	5	−3
Homebaking, jam, spreads	1849	5	3
Fruit and vegetables	1652	4	14
Frozen foods	1419	4	−1
Canned/tinned foods	1312	3	2
Drinks-fruit juice	1171	3	14
Breakfast cereals	745	2	7
Drinks-other	620	2	6
Specialty/ethnic foods	615	2	11
Drinks—soft	496	1	18
Fish	421	1	11
Oils	314	1	−5
Drinks-hot	308	1	17
Total	38,417	100	

## Appendix A

**Table A1:** Examples of mapping decisions made to match generic food codes to branded foods using food description and “Back of Pack data”.

Food Item	Mapping Decision
Artificial sweeteners and sugar substitutes	Macronutrients taken from BOP and micronutrients mapped to “White sugar”
Isotonic, sports and energy drinks (i.e., Red Bull, Powerade)	All Lucozade products mapped to “Lucozade” For other energy drinks, macronutrients taken from BOP and micronutrients mapped to “Fruit juice drink, carbonated, ready to drink”
Energy tablets, bars and snacks (i.e., “Lucozade Energy Original Glucose Tablets”, “Lucozade Sport Body Fuel Carbohydrate Energy Mixed Berry Flavour Cereal Bar”)	Glucose tablets—Macronutrients BOP, micronutrients mapped to “Glucose liquid” Cereal based energy bars such as “Lucozade Sport Body Fuel Carbohydrate Energy Mixed Berry Flavour Cereal Bar” mapped to “cereal, chewy bar” Where there was no appropriate item to map to, items were mapped on an individual basis as a recipe, guided by the ingredient list. For example, “Science in Sport Go Nutritional Energy Bar Banana Fudge Flavoured” was mapped to 35% “Fruit juice drink, ready to drink”; 16% “Dates, dried; 10% bananas; 10% “rice krispies”; 10% “soya flour, low fat” 8% “oatmeal, raw”; 6% raisins; 5% “apricots, dried”
Vegetarian food products (Whilst “Quorn, pieces, as purchased” is listed in McCance and Widdowson v.6, it is limited when attempting to capture the range of different quorn products such as “Quorn Bacon Style Rashers”	All quorn products mapped to “Quorn, pieces, as purchased” “Vegetarian sausages, baked/grilled” used specifically for vegetarian sausages
Blueberries (blueberries are available in the myfood24 database from a manufacturer called “Ardo” but there are no generic blueberry options in the McCance and Widdowson (v.6) food tables	There is no generic code for blueberries in the myfood24 database. Users can select the “Ardo” blueberries code. The macronutrients are from BOP and the micronutrients have been mapped to bilberries which offered the most similar nutrient information.
Perry (Considered to be a pear cider but is not legally recognised as such due to its high sugar content)	Macronutrients from BOP and micronutrients mapped to “Cider, sweet” (from apples)
Sake	Mapped to white wine, dry
Flavoured vodka and “alcopops” e.g.,: “Smirnoff ice” and “Barcardi Breezer”	Mapped to 10% “spirits”, 90% “fruit juice drink carbonated”
Schnapps	Mapped to fortified wine “port” which has a similar alcohol content (15%–20%)
Egg free mayonnaise	Micronutrients mapped to “ Mayonnaise, retail” (macronutrients from BOP)
1% fat milk	Macronutrients from BOP. Micronutrients mapped to “ Skimmed milk, average”
Goats milk cream and yogurt (whilst there are options for goats cheese and milk there are no specific codes for other goat products)	Macronutrients from BOP and other goat products mapped to dairy products from a cow
Best of both bread and bread products	Macronutrients from BOP. Micronutrients mapped to 50% white bread and 50% wholemeal bread
Carbonated drinks: dandelion and burdock, tonic water and sugar-free soft drinks.	Macronutrients from BOP. Micronutrients mapped to “diet, cola”
Meal replacements	Macronutrients from BOP. Micronutrients for milk based meal replacements mapped to “Flavoured milk, pasteurised” Meal replacement bars mapped to “Cereal chewy bar”
Garlic bread (The M&W code is “Garlic bread, pre-packed, frozen”) but does not account for different types of bread.	For garlic bread with different kinds of bread this has been mapped as a recipe. For example: “Tesco finest garlic ciabatta slices mapped to 98% ciabatta and 2% garlic puree”
Vegetable juice (Codes for carrot juice and tomato juice but no code for “vegetable juice”)	Mapped as a recipe following ingredient list. For example: “V8 Vegetable Juice Original” mapped to: 87% tomato juice; 5% carrot juice; 2% celery, raw; 2% beetroot, raw; 2% parsley fresh; 2% watercress, fresh; 1% spinach, canned and drained as per ingredients list.
Seasonal products (these have a very short shelf life and are likely to be different from year to year)	Most seasonal products removed, some kept (although likely to change at least there might be something similar to select). There are currently; 65 Christmas products, 8 Halloween products, 50 Easter eggs, 3 Valentine’s day chocolate products
High protein milk based drinks, i.e., “Maximuscle High Protein”	Macronutrients from BOP and micronutrients mapped to “Build-up powder, shake”
Cordial	Cordials mapped to the undiluted cordials or made-up to offer choice. For example the generic codes used primarily were “Lime juice cordial, undiluted” or “Fruit juice drink, low calorie, ready to drink”. Descriptors in the portion estimation screen have been added to clarify whether the selection is for concentrated or diluted product.
Powder based foods (custard, angel delight, gravy, stock cubes)	Powders were mapped to powders and the portion options presented as per pack instructions and clarified as powder. For example: Bird’s Instant Custard mapped to “custard powder” and serving size presented as: 75 g per packet (as powder). This was done because it’s not known whether the person would make up the product with milk or with water. There are also versions of the items “as consumed” for the user to select. Descriptors in the portion estimation screen have been added to clarify whether the selection is for dry or wet product. Gravy was mapped to granules, for example: “Bisto Best Lamb Gravy with a Hint of Mint” mapped to “gravy instant, granules” and serving option was as per BOP: 30 g per packet or 5 g teaspoons. Angel delight mapped to “instant dessert powder”: serving option as per pack: 30 g packet (dry)
Cake mix, biscuit mix	Mapped to the finished product and serving size given as per finished product. For example, Dr. Oetker Halloween Cupcake Kit mapped to “chocolate cake”. Portion options in myfood24 are “as served” per cake slice. Betty Crocker Double Choc Chip Cookie Mix mapped to “cookies, chocolate chip” and serving is per 29 g cookie.
Raw and dried foods	Raw and dried products (such as dried pasta and rice) have been mapped to a generic cooked alternative and cooking factors (from the UK food composition tables version 7) applied to account for volume change and nutrient loss attached to each particular item.
Vitamin and mineral fortified products	Individual changes made to the mapping to reflect modified amounts where products have been fortified. For example, “Tropicana Essentials Vitamin A Plus Antioxidants C and E Drink” has been mapped to a generic fruit juice code and vitamins A, C and E have been manually adjusted based on the manufacturer data. The item is therefore be an amalgam of the 8 BOP macronutrients, generic micronutrients and manually adjusted ACE vitamins.
Dairy free products (52 dairy free products)	Macronutrients from BOP but micronutrients mapped to most appropriate dairy containing equivalent. i.e.,: dairy free white chocolate buttons mapped to “chocolate, white”
Gluten free products (243 gluten free products)	Where product is labelled as gluten free but it is not likely to differ significantly from gluten containing equivalent then macronutrients were taken from BOP but micronutrients mapped to gluten containing equivalent, for example: “Nairns gluten free oatcakes” mapped to “oatcakes”. Where product contains a lot of gluten and likely to be very differently formulated then broken down and mapped as a recipe by ingredient list, for example: “DS Gluten Free Breadsticks” mapped as: 30% potato flour; 20% rice flour; 20% cornflour; 15% buckwheat; 6% yeast, dried; 5% vegetable oil, blended, average; 2% sugar white; 2% salt.
Canned vegetables (In M&W the nutrient values are for the proportion of edible contents after liquid has been drained off, except where otherwise stated i.e., tomatoes, canned, whole contents)	For example: Batchelors Mushy Peas mapped to “Mushy peas, canned, re-heated”
Canned fruit (In M and W, nutrient values for canned fruit includes syrup and juice, unless otherwise stated. M&W does not give the nutrient values for only the fruit consumed)	For canned fruit only generic options are presented in the myfood24 FCDB
Canned tuna (In M&W the nutrient values are for the proportion of edible contents after liquid has been drained off. M&W has two codes: “Tuna, canned in brine, drained” or “Tuna, canned in oil, drained”)	For example: “Tesco everyday value tuna chunks in brine” was mapped to “Tuna, canned in brine, drained” as the BOP nutrient values are for drained content. However, for a couple of products which were specific “no drain tuna” the macronutrients would be BOP but micronutrients would come from drained equivalent
